# Hyperdiploidy associated with T315I mutation in BCR-ABL kinase domain in an accelerated phase-chronic myeloid leukemia case

**DOI:** 10.1186/s13039-014-0089-0

**Published:** 2014-11-29

**Authors:** Walid Al-Achkar, Faten Moassass, Adnan Ikhtiar, Thomas Liehr, Moneeb Abdullah Kassem Othman, Abdulsamad Wafa

**Affiliations:** Human Genetics Division, Department of Molecular Biology and Biotechnology, Atomic Energy Commission, P.O. Box 6091 Damascus, Syria; Mammalians Biology Division, Department of Molecular Biology and Biotechnology, Atomic Energy Commission, Damascus, Syria; Institute of Human Genetics, Jena University Hospital, Jena, Germany

**Keywords:** Chronic myeloid leukemia, Philadelphia chromosome, Hyperdiploidy, T315I mutation, Prognostic factors

## Abstract

**Background:**

Chronic myeloid leukemia (CML) is genetically characterized by the occurrence of a reciprocal translocation t(9;22)(q34;q11), resulting in a BCR/ABL gene fusion on the derivative chromosome 22, i.e. the Philadelphia (Ph) chromosome. During CML progression 60–80% of the cases acquire additional genetic changes. Even though hyperdiploidy is not a rare finding in advanced phase-CML, hyperdiploidy together with a T315I kinase domain (KD) mutation in the BCR-ABL gene has not yet been reported.

**Results:**

A complete cytogenetic and molecular cytogenetic analysis; molecular biology methods such as quantitative reverse transcription polymerase chain reaction (RQ-PCR) and allele-specific oligonucleotide (ASO)-PCR; and immunophenotypically confirmed CML in acceleration phase (AP). Our case revealed the presence of hyperdiploidy including multiple copies of the Ph chromosome, presence of b3a2 fusion transcript,T315I mutation in BCR-ABL KD in pre imatinib mesylate (IM) treatment. The ratio of BCR-ABL/ABL expression in post nilotinib treatment was 0.07% on international scale.

**Conclusions:**

The patient demonstrated a good response to nilotinib after imatinib failure; while the hyperdiploid clone disappeared the T315I mutation remained during follow-up. The underlying mechanisms and prognostic implications of these cytogenetic abnormalities are discussed.

## Background

Chronic myeloid leukemia (CML) is a disorder characterized by the formation of granulocytes in connection with a translocation involving chromosomes 9 and 22. Resulting derivative chromosome 22 is called Philadelphia chromosome (Ph) [1]. A simple reciprocal translocation of t(9;22)(q34;q11) can be observed as single aberration in the early stage chronic phase (CP) whereas in most advanced cases this change is accompanied by other chromosomal aberrations. In fact, a chimeric gene BCR-ABL gene and protein is formed. The fused gene has an oncogenic activity and encodes for an active tyrosine kinase [2]. Hyperdiploidy is not common in CML cases [3] and it is a common finding in advanced phase-CML patients [4,5] and it was already in one CML-CP patient as a secondary chromosomal aberration after imatinib mesylate (IM) therapy [6].

Imatinib (IM = Glivec, formerly STI571) is a chemically designed drug which blocks BCR/ABL1 tyrosine kinase activity and is successfully used in CML patients [7]. Nilotinib is a second generation tyrosine kinase inhibiter (TKI) with improved target specificity and potency. Nilotinib acts like IM and binds to the kinase domain (KD) of *ABL1*. This prevents the formation of an active conformation, catalyzing the transduction of the BCR-ABL signal [8]. Nilotinib was approved for CML treatment in CP and AP especially in cases showing IM resistance due to mutations in BCR-ABL tyrosine kinase geneor intolerance to prior successful IM therapy [9,10]. T315I is one of the most frequent mutations in the BCR-ABL domain and has been associated with TKI resistance of 1st and 2nd generation drugs [11].

Here we presented a new CML case in AP pre IM treatment with hyperdiploidy including more than one copy of the Ph chromosome, presence of b3a2 fusion transcript, a T315I mutation in the BCR-ABL KD, and a ratio of BCR-ABL/ABL1 expression post nilotinib treatment was 0.07% on international scale (IS). This patient demonstrated a good response to nilotinib after imatinib failure.

## Case presentation

In June 2012 a 53-year-old male was diagnosed as suffering from CML. Physical examination revealed hepatosplenomegaly, several skin nodules (2 cm) in different locations such as neck and armpit (data not shown), anemia, thrombocytopenia, fever, fatigue, and loss of weight were the indicative symptoms. The patient’s hematologic parameters were white blood cells (WBC) of 52.2x10^9^/l (15% of cells were blasts), red blood cell (RBC) count was 2.50x10^6^/mm^3^, hemoglobin level was 7.1 g/dl and the platelet count was 90x10^9^/l. Serum lactate dehydrogenase value (LDH) was 1,851 U/l (normal level <460 U/l). The patient was diagnosed as CML-AP according to WHO recommendations and in an intermediate Sokal risk of 0.89 (0.8-1.2). No treatment had been administered prior to the test.

The patient was referred for a second time in November 2013 and was treated with IM (400 mg/day) for 5 months. Thus therapeutic scheme was changed to nilotinib (600 mg/day) for 6 months with disappeared the previous reported relevant symptoms. The more recent hematological parameters were: WBC 5.3x10^9^/l (50.2% neutrophils, 48.1% lymphocytes, 0.9% monocytes and 0.8% eosinophiles). The platelet count was 118x10^9^/l and the hemoglobin level was 12.4 g/dl.

## Results

**Figure 1:**
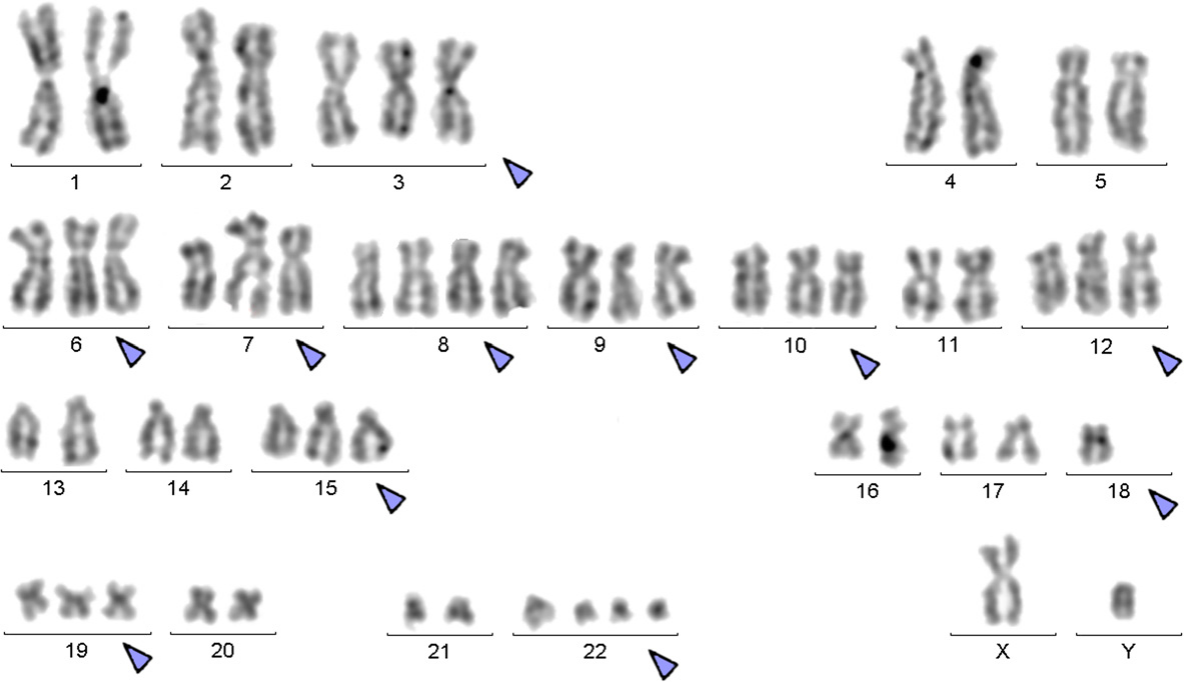
**GTG-banding revealed a hyperdiploid karyotype.** All derivative chromosomes are shown and with arrow.

**Figure 2:**
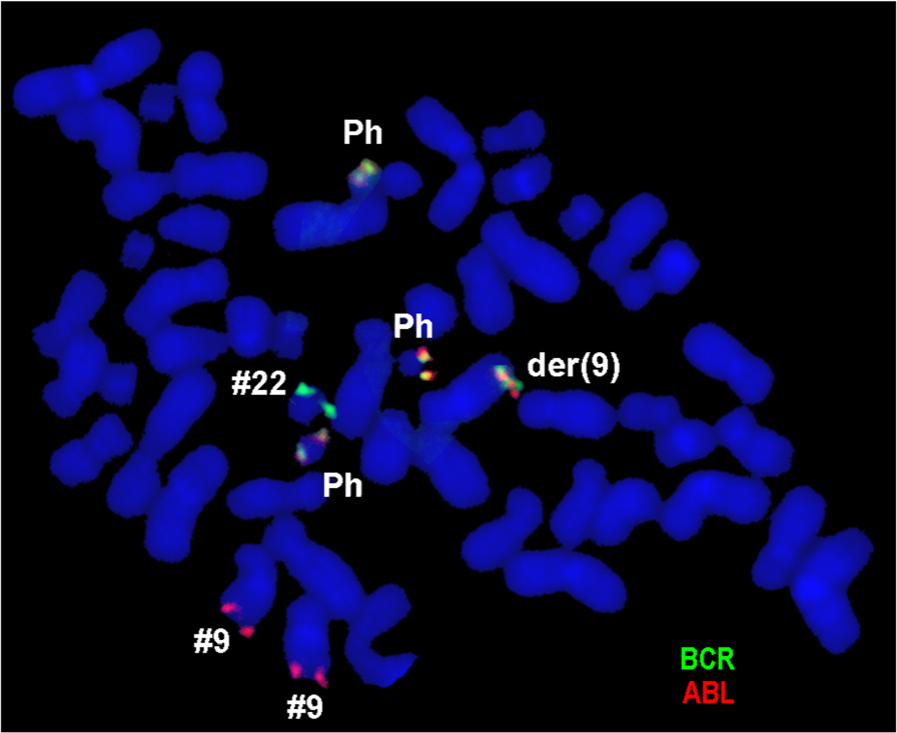
**Partial metaphase FISH using probes for BCR (green) and ABL (red) showed 4 copies of BCR/ABL in this case, three copies on Ph chromosome and one on der(9).** Abbreviations: # = chromosome; der = derivative chromosome.

**Figure 3:**
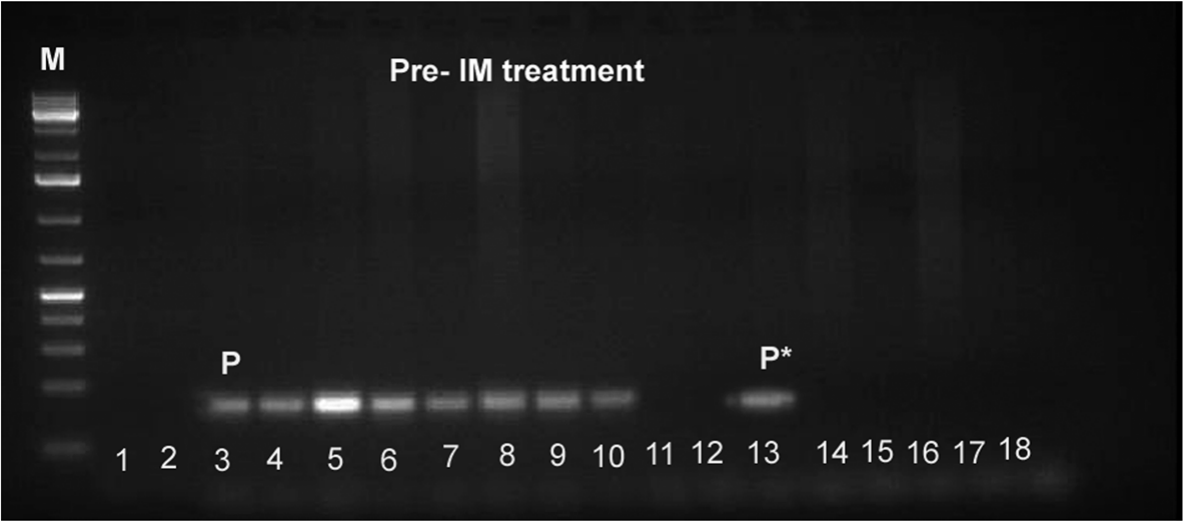
**ASO-PCR monitoring and corresponding sensitivity test for detected T315I mutation pre-IM treatment.** ASO-PCR products on genomic DNA obtained from healthy donor and our patient. Line M, 1 kb DNA ladder; lines 1,2 blank; lines 3–10 patients ASO-PCR T315I wild-type primers; P, our patient; lines 11–18 ASO-PCR T315I mutated primers; and P*, our patient with T315I mutation.

**Figure 4:**
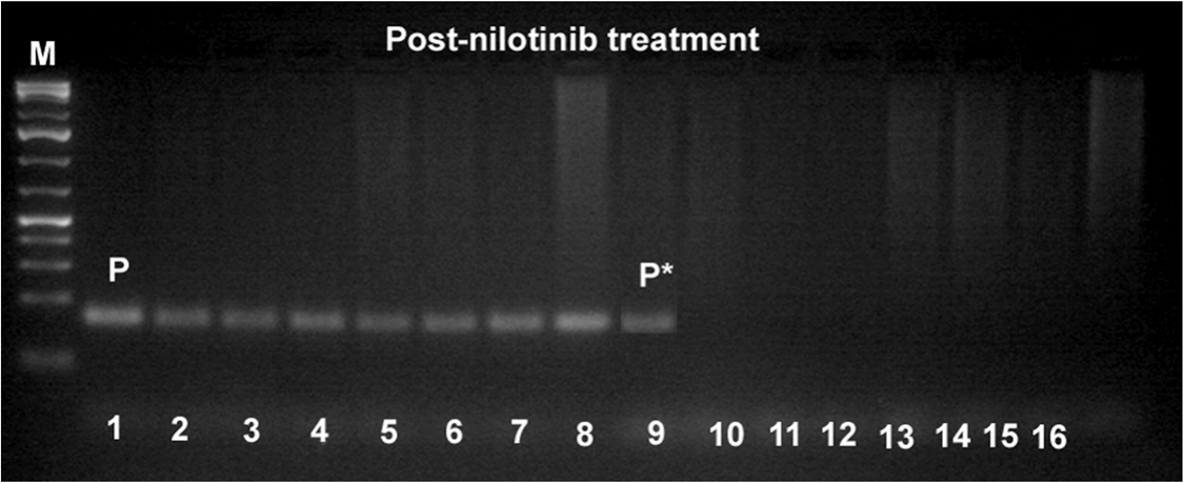
**ASO-PCR monitoring and corresponding sensitivity test for detected T315I mutation Post-nilotinib treatment.** Line M, 1 kb DNA ladder; lines 1–8 patients ASO-PCR T315I wild-type primers; P, our patient; lines 9–16 ASO-PCR T315I mutated primers; and P*, our patient with T315I mutation.

Pre IM treatment banding cytogenetics revealed a karyotype of 46,XY,t(9;22)[7]/56,XY,+3,+6,+7,+8,+8,+9,t(9;22),+10,+12,+19,+der(22)t(9;22)[5]/57,XY,+3,+6,+7,+8,+8,+9,t(9;22),+10,+12,+15,-18,+19,+der(22)t(9;22)*x*2[8]. Post nilotinib treatment banding cytogenetics revealed a karyotype of 46,XY,t(9;22)[1]/46,XY[39] (Figure 1). Further studies were done based on molecular cytogenetics (Figure 2) and molecular genetics (Figure 3). Dual-color-FISH pre IM treatment; using a specific probe for BCR and ABL1 revealed onefusion signal on the derivative chromosomes 9 [der(9)] and another three fusion signals on the up to three Ph chromosomes (Figure 2); and chromosomes 3, 6, 7, 8, 9, 10, 12, 15, 19 and 22, were studied with WCP and/or CEP probes and did not provide any hint on cryptic translocations (data not shown). RT-PCR pre IM treatment and post nilotinib treatment confirmed the presence of the BCR-ABL1 fusion (b3a2 transcript) revealing a major M-BCR transcript (data not shown). RQ-PCR post nilotinib treatment demonstrated a ratio of BCR-ABL/*ABL1* expression of 0.07% on IS (data not shown). ASO-PCR pre IM treatment and post nilotinib treatment results showed the presence of the T315I mutation (Figure 3, (Figure 4). The final karyotype pre IM treatment and post nilotinib treatment was determined:

Pre IM treatment : 46,XY,t(9;22)(q34;q11.2)[7]/56,XY,+3,+6,+7,+8,+8,t(9;22)(q34;q11.2),+10,+12,+19,+der(22)t(9;22)(q34;q11.2)[5]/57,XY,+3,+6,+7,+8,+8,t(9;22)(q34;q11.2),+10,+12,+15,-18,+19,+der(22)t(9;22)(q34;q11.2)*x*2[8], and post nilotinib treatment: 46,XY,t(9;22)(q34;q11.2)[1]/46,XY[39].

The abnormal cell population (~19% blasts in peripheral blood specimen) showed the following immunophenotype, which was consistent with CML-AP (WHO recommendations): CD45^+^, CD34^+^, CD10^+^, CD19^−^, CD20^−^, CD22^−^, CD5^−^ and CD7^−^, HLADr^−^, CD33^+^, CD32^+^, CD16^−^, CD117^−^, CD14^−^, CD15^−^, CD64^−^ and expressed CD11c, CD38, CD235a and CD13 heterogeneously.

## Conclusions

Presence of multiple copies of the Ph chromosome is one of several types of IM resistance mechanisms [12,13].

According to the literature, there are a few near triploid karyotypes reported in advanced phase-CML [14,15]. Stagno et al. [2] found a CML case in blast phase with hyperdiploidy and it includes 10% mutant clones with V256G mutation in BCR-ABL domain and e13a2 BCR-ABL transcript. In this paper, we described a new CML case in AP with hyperdiploidy including multiple copies of Ph chromosome, presence of b3a2 fusion transcript and T315I mutation in the BCR-ABL KD. To the best of our knowledge, these chromosomal abnormalities, particularly hyperdiploidy associated with T315I mutation have not been previously observed in CML [16].

Approximately 10% of CML patients present in advanced phases called CML-AP or blast crisis (CML-BC), without a clinically evident CP [17]. Even with the advent of TKI therapy, the phase of disease remains an important prognostic factor in CML [18,19].

Disease progression is uncommon currently in CML patients treated in CP with TKI. When it occurs, progression usually occurs early during treatment. 3.3% of CML-CP patients progressed to CML-AP/CML-BC within the first 18 months of therapy, while <1% progressed after 4 years of therapy [20]. The criteria for CML-AP in patients progressing on TKI therapy are still poorly defined and disease progression in CML is currently defined more practically in terms of responsiveness to TKI therapy [16].

The progression to advanced phases of disease is caused by the development of genetic lesions in addition to BCR-ABL; secondary cytogenetic changes are identified in 50-80% of CML- BC cases [21]. Also this phenomenon was described in 5-10% of patients presenting with CML-CP and in 30% of patients developing AP features, and has overall been associated with poor prognosis [22,23]. Cytogenetic clonal evolution was subsequently considered as one of the factors defining CML-AP [24].

The most common mechanism of secondary resistance to TKI is mutation in the KD of BCR-ABL. These KD mutations develop as a result of selection pressure and correlate with progression to advanced phases and shortened survival. Thus, early identification of IM resistance is critical to allow the use of other, potentially effective therapies, such as second generation TKI and/or stem cell transplantation [25].

The mutation at amino acid 315 in the imatinib-binding site (T315I mutation) confers resistance to imatinib, dasatnib, and nilotinib by preventing access of these drugs to the ATP-binding pocket [26-28]. Hughes et al. [29] found six cases (5 IM resistant and one IM intolerant, i.e. six in total) with T315I mutation and two of the six cases did not showed responses to Nilotinib but couldn’t sustain. In addition, Jabbour et al. [30] found one case of CML-AP with T315I mutation and complete cytogenetic response to Nilotinib. Also, Jabbour et al. [31] found of CML-AP with T315I mutation and Nilotinib resistance. However, T315I mutation also was observed in 10 cases post IM failure (4 CP, 5 AP, and 1 blast phase) and 2 cases post Nilotinib treatment in the study by Cortes et al. [32]. Cea et al. [33] also found T315I mutation post IM, post Nilotinib and during Dasatinib treatment in e19a2 BCR/ABL1 CML. Our patient achieved a major cytogenetic response and major molecular response after 6 months of nilotinib treatment with T315I mutation remaining.

The mechanisms by which these mutations develop are poorly understood. KD mutations can be present at low-levels in CML cases prior to exposure to TKI therapy. However, these small mutant clones do not necessarily confer an adverse prognosis and resistant clones may not expand even under the selection pressure of TKI therapy [34,35]. The KD mutations usually occur within the first 2 years of TKI therapy and are much more common in CML-AP and CML-BC than CML-CP [7].

In conclusion, we described a new CML case in AP pre IM treatment with hyperdiploidy including multiple copies of Ph chromosome, and presence of T315I mutation in the BCR-ABL KD. The patient demonstrated a good response to nilotinib treatment after imatinib course failure. Interestingly hyperdiploidy clone disappeared while the T315I mutation remained.

## Materials and methods

### Chromosome analysis

Chromosome analysis applying GTG-banding according to standard procedures [36] was performed pre IM treatment and post nilotinib treatment. 20 metaphase cells derived from unstimulated bone marrow culture were analyzed. Karyotypes were described according to the International System for Human Cytogenetic Nomenclature (ISCN 2009).

### Molecular cytogenetics

Fluorescence in situ hybridization (FISH) using the LSI BCR/ABL dual color dual fusion translocation probe (Abbott Molecular/Vysis, Des Plaines, IL, USA) and/or chromosome enumeration probes (CEP) for chromosomes 6, 7, 8 and/or 12 (Abbott Molecular/Vysis, Des Plaines, IL, USA) were applied together with whole chromosome painting (WCP) probes for chromosomes 3, 9, 10, 15, 19 and/or 22 (MetaSystems, Altlussheim, Germany) according to manufacturer’s instructions [36]. A minimum of 10 metaphase spreads was analyzed, using a fluorescence microscope (AxioImager.Z1 mot, Carl Zeiss Ltd., Hertfordshir, UK) equipped with appropriate filter sets to discriminate between a maximum of five fluorochromes plus the counterstain DAPI (4′,6- diamino-2-phenylindole). Image capture and processing were performed using an ISIS imaging system (MetaSystems, Altlussheim, Germany).

### Reverse transcriptase-polymerase chain reaction (RT-PCR) and quantitative reverse transcription polymerase chain reaction (RQ-PCR) for BCR/ABL1 fusion transcripts

Total RNA extracted from peripheral blood sample using the InviTrap RNA kit (Invitek, Berlin, Germany) according to the manufacturer’s recommendations. cDNA was prepared from 5 μg of total RNA with the Genequality BCR-ABL1 kit (AB Analitica, Padova, Italy) and BCR-ABL1 fusion transcript was performed according to the manufacturer’s instructions (AB Analitica, Padova, Italy). RQ-PCR analyses were performed in StepOne Real-Time PCR (Applied Biosystems, Foster City, California, USA) using one-step qRT-PCR BCR-ABL1 kit (MolecularMD, Portland, USA) according to the manufacturer’s recommendations.

### Allele-specific oligonucleotide (ASO)-PCR

Mutated or wild-type sequences were amplified in a noncompetitive PCR reaction performed on genomic DNA and PCR conditions using allele-specific and reverse primers for the Thr315Ile mutation as described previously. The length of the PCR product using 1 Kb DNA ladder (Fermentas, Lithuania, Vilnius) was (158 bp). The sensitivity of this assay was determined for each mutation by amplification of 10-fold limited dilutions of 100 ng patient’s DNA at time of resistance in 100 ng healthy control DNA [37].

### Flow cytometricimmunophenotype

Flow cytometry of leukemic blasts was performed using a general panel of fluorescent antibodies against the following antigens typical for different cell lineages and cell types: CD1a, CD2, CD3, CD4, CD5, CD8, CD10, CD11b, CD11c, CD13, CD14, CD15, CD16, CD19, CD20, CD22, CD23, CD32, CD33, CD34, CD38, CD41a, CD45, CD56, CD57, CD64, CD103, CD117, CD123, CD209, CD235a and CD243; in addition antibodies against Kappa and Lambda light Chains, sIgD, sIgM, and HLADr were applied (BD Biosciences). Four-color immunophenotyping on peripheral blood specimen was performed. Samples were stained and analyzed on a BD FACSCalibur™ flow cytometer according to BD Biosciences manuals and products insert sheets. Autofluorescence, viability, and isotype controls were included. Flow cytometric data acquisition and analysis were conducted by BD Cellquest™ Pro software.

## Consent

Written informed consent was obtained from the patient for publication of this Case Report. A copy of the written consent is available for review by the Editor-in-Chief of this journal.
